# Rapid one-step enzyme immunoassay and lateral flow immunochromatographic assay for colistin in animal feed and food

**DOI:** 10.1186/s40104-019-0389-7

**Published:** 2019-10-17

**Authors:** Jiayi Wang, Jinyu Zhou, Yiqiang Chen, Xinpei Zhang, Yongpeng Jin, Xiaojing Cui, Dongting He, Wenqing Lai, Lidong He

**Affiliations:** 1 Beijing Advanced Innovation Center for Food Nutrition and Human Health, and State Key Laboratory of Animal Nutrition, College of Animal Science and Technology, China Agricultural University, Beijing, China; 2 Department of Chemistry and Biochemistry, Florida State University, Tallahassee, FL USA

**Keywords:** Colistin, ELISA, Feed, Food, Gold nanoparticle, Lateral flow immunochromatographic assay, Monoclonal antibody

## Abstract

**Background:**

Colistin (polymyxin E) is a kind of peptide antibiotic which has been approved in animal production for the purposes of disease prevention, treatment, and growth promotion. However, the wide use of colistin in animal feed may accelerate the spread of colistin-resistance gene MCR-1 from animal production to human beings, and its residue in animal-origin food may also pose serious health hazards to humans. Thus, it is necessary to develop corresponding analytical methods to monitor the addition of colistin in animal feed and the colistin residue in animal-origin food.

**Results:**

A one-step enzyme-linked immunosorbent assay (ELISA) and a lateral flow immunochromatographic assay (LFIA) for colistin were developed based on a newly developed monoclonal antibody. The ELISA showed a 50% inhibition value (IC_50_) of 9.7 ng/mL with assay time less than 60 min, while the LFIA had a strip reader-based detection limit of 0.87 ng/mL in phosphate buffer with assay time less than 15 min. For reducing the non-specific adsorption of colistin onto sample vial, the components of sample extraction solution were optimized and proved to greatly improve the assay accuracy. The spiked recovery experiment showed that the recoveries of colistin from feed, milk and meat samples were in the range of 77.83% to 113.38% with coefficient of variations less than 13% by ELISA analysis and less than 18% by LFIA analysis, respectively. Furthermore, actual sample analysis indicated that the two immunoassays can produce results consistent with instrumental analysis.

**Conclusions:**

The developed assays can be used for rapid qualitative or quantitative detection of colistin in animal feed and food.

**Electronic supplementary material:**

The online version of this article (10.1186/s40104-019-0389-7) contains supplementary material, which is available to authorized users.

## Background

Colistin (polymyxin E) is a kind of peptide antibiotic which is produced by *Bacillus polymyxa* subsp. it is generally a mixture that can be divided into colistin A (polymyxin E_1_) and colistin B (polymyxin E_2_) depending on the fatty acid side chain. In human clinic, colistin is used to treat infections from multidrug-resistant Gram-negative bacteria such as Enterobacteriaceae and *Pseudomonas aeruginosa.* Because it is considered one of the last-resort antibiotics for these infections [1], the World Health Organization [2] has classified it in the category of critically important antimicrobials. In the meantime, colistin has been approved to be used in animal production for the purposes of disease prevention, treatment, and growth promotion [3]. However, after the use of colistin in animal production for decades, a significant resistance of Enterobacteriaceae to colistin in livestock has been reported by several research groups [4–6]. More recently, Shen and his colleagues identified the first mobile colistin-resistance gene named MCR-1 in *Escherichia coli* which was isolated from food-producing animals [7], and this gene was subsequently reported in more than 30 countries [8]. These studies suggested that addition of colistin in animal diet has accelerated the spread of MCR-1 from animal production to human beings [9]. After extensive risk assessment, the Chinese Ministry of Agriculture released an announcement (No. 2428) to ban the use of colistin as a feed additive to animals since April of 2017 [10]. Moreover, colistin as a veterinary pharmaceutical remains legal use to treat intestinal infections in animals and cow mastitis. Nevertheless, the intramuscular or intramammary administration of colistin may lead to colistin residue in edible food such as milk and meat, and subsequently pose potential nephrotoxicity and neurotoxicity to human. Meantime, the low level of colistin in animal-origin food may also induce resistant bacteria as the predicted no effect concentrations of colistin for resistance selection is only 2 ng/mL [11]. To maintain consumer health, the maximum residue limits (MRLs) of colistin in edible food such as milk and meat have been set at 50 and 150 μg/kg respectively by the Codex Alimentarius FAO-WHO [12]. Therefore, it is necessary and important to monitor the illegal addition of colistin in animal feed and the colistin residue in animal-origin food for combating bacteria drug-resistance and ensuring public health.

Analytical methods for colistin measurement include microbiological assay [13, 14], enzyme immunoassay [15], high performance liquid chromatography (HPLC) [16–19], and liquid chromatography coupled with tandem mass spectrometry (LC-MS/MS) [20–24]. Microbiological assay is relatively labor-insensitive and time-consuming compared to these methods [15, 25] and is now rarely used for colistin detection. HPLC analysis requires complex sample pretreatment such as protein precipitation and solid phase extraction. Moreover, as colistin has no strong ultraviolet (UV)-absorption and fluorescence, it has to be derivatised with 9-fluorenylmethyl chloroformate (FMOC-Cl) [16, 19] or ortho-phthalaldehyde (OPA) [17, 18] before UV or fluorescence detection. LC-MS/MS has been widely used for the determination of colistin in different matrixes owing to its high sensitivity and high selectivity, but it requires even more extensive sample pretreatment and is more susceptible to sample matrixes than LC analysis [23, 24]. Therefore, these two types of instrumental analysis are not advisable for rapid screening of bulk samples. As a comparison, immunoassay such as ELISA and LFIA has the advantages of high assay sensitivity, high throughput and rapid turnaround time, making them more suitable for rapid monitoring of colistin [26]. Previously, Kitagawa et al. [25] reported an enzyme immunoassay for colistin in rainbow trout tissue, but the assay sensitivity was too low to be practically used. Suhren and Knappstein [15] developed an ELISA approach for colistin in milk, but polyclonal antibodies were employed in the study, the titers and affinities of each batch of sera would be different. Moreover, the accuracy and precision of this assay was not good enough for quantitative analysis [15]. Therefore, in this study, we prepared a new monoclonal antibody against colistin and developed a rapid one-step ELISA for colistin in animal feed and food. As compared to ELISA, LFIA is a more rapid, simpler and more economic approach and is suitable for the detection of colistin in field environment. Currently, there is no research report about LFIA for colistin in biological matrixes. Thus, based on the prepared mAb, we also developed a LFIA as an alternative to ELISA for on-site detection of colistin. In addition, one obstacle for accurate determination of colistin in biological matrixes relies on its non-specific adsorption on container surface, which has been extensively reported in the development of instrumental analysis [23, 27]. Internal standard such as polymyxin B was generally employed to counteract the negative effect of non-specific adsorption [23, 28]. However, the use of polymyxin B as internal standard is not appropriate in immunoassay since the molecular structures between polymyxin B and colistin are quite similar and the antibody against colistin would also recognize polymyxin B. Thus, in this study, we investigated the extent of non-specific adsorption on different sample vials and optimized the extraction solution, aiming to reduce non-specific adsorption of colistin and improve the assay accuracy and precision.

## Methods

### Chemicals and apparatus

Colistin sulfate, kanamycin sulfate, neomycin sulfate, streptomycin sulfate, gentamicin sulfate, 1-ethyl-3-(3-dimethylaminopropyl) carbodiimide (EDC), chlorauric acid, bovine serum albumin (BSA), ovalbumin (OVA), Freund’s complete adjuvant, Freund’s incomplete adjuvant, goat anti-mouse IgG and goat anti-mouse IgG-horseradish peroxidase (HRP) conjugate were bought from Sigma-Aldrich (St. Louis, MO, USA). RPMI 1640 medium, fetal bovine serum (FBS), polyethylene glycol 1500 (PEG 1500), hypoxanthine-aminopterin-thymidine (HAT) medium, and hypoxanthine-thymidine (HT) medium were supplied by Gibco BRL (Rockville, MD, USA). Other reagents were provided by Beijing Regent Corporation (Beijing, China).

Swine feed and chicken feed used as blank matrixes are obtained from Da Bei Nong Group (Beijing, China) and New Hope Liuhe Group (Beijing, China). Nitrocellulose membrane CN 140 was bought from Whatman International Ltd. (Middlesex, UK). Absorbent pad CH 37, adhesive backing card and sample pad (GF2-II) were provided by Shanghai GoldBio Co. Ltd. (Shanghai, China). Microtiter plates, cell culture bottles, microculture plates, were bought from Costar Group Inc. (Bethesda, MD, USA). HM 3030 Dispensing Platform and ZQ 2000 Guillotine Cutting Module (Shanghai GoldBio Co. Ltd., Shanghai, China) were used for the assembly of LFIA strips.

### Preparation of immunogen and coating antigen

The conjugates colistin-BSA and colistin-OVA were synthesized by carbodiimide coupling method according to the procedure as below. Briefly, 10 mg of carrier protein (BSA or OVA) was dissolved in 2 mL of 0.05 mol/L MES buffer (pH 6.0, 0.5 mol/L NaCl) and then 0.5 mL of 40 mg/mL EDC was added dropwise. After reaction at room temperature for 2 h, the colistin-protein conjugates were dialyzed against phosphate buffer saline (PBS) at 4 °C for 2 days and were then characterized by MALDI-TOF mass spectrometry (Bruker Daltonics, Germany). The colistin-BSA conjugate was used as immunogen and the colistin-OVA conjugate was used as coating antigen.

### Preparation of monoclonal antibody (mAb)

#### Immunization

The procedures used throughout this experiment were approved by the China Agricultural University Institutional Animal Care and Use Committee (CAll20160925–1, Beijing, China). Twenty 8 weeks old female BALB/c mice were divided into two groups (10 mice each) and immunized with colistin-BSA conjugate. The mice in the two groups were respectively immunized with 15 μg or 60 μg of the immunogen per mouse by subcutaneously injecting the emulsion of colistin-BSA and Freund’s complete adjuvant. Then three booster immunizations were performed at every 4 weeks and the immunization dosage was the same but the immunogen was emulsified in Freund’s incomplete adjuvant. Seven days after each immunization, the antisera were collected and evaluated for anti-colistin activity by competitive indirect ELISA (ci-ELISA). The mouse producing antiserum with the highest affinity to colistin received another immunization intraperitoneally, and 4 days later, the mouse was sacrificed for cell fusion and hybridoma production.

#### Cell fusion and hybridoma production

Cell fusion was performed according to the following procedure [29]. Briefly, the spleen lymphocytes of the sacrificed mouse was fused with myeloma cells at a ratio of 5:1 using PEG 1500 as fusing reagent. The fused cells were then suspended in HAT-RPMI 1640 medium containing 20% fetal calf serum and 10 μg/mL gentamicin and streptomycin, and were then allocated to ten 96-well microculture plates. Twelve days following the fusion, the supernatants of each well were tested by both noncompetitive and competitive indirect ELISA. The cells in micro-culture wells indicating a significant colistin recognition activity were further cloned by limiting dilution using HT-RPMI 1640 medium containing 20% fetal calf serum. The cell clones that can stably produce antibodies were then large-scale cultured and finally cryopreserved in liquid nitrogen.

#### Production and purification of monoclonal antibody (mAb)

Mature female BALB/c mouse was intraperitoneally injected with 0.5 mL of paraffin. Eight days later, the antibody-producing hybridoma cells was intraperitoneally injected. After 11 days, ascites fluid can be collected and the purification of mAb was performed by saturated ammonium sulfate method followed by passing through Hitrap protein A HP antibody purification column (GE Healthcare Life Sciences, USA). The isotypes of the purified mAbs were determined by a rapid mouse antibody isotyping ELISA kit (Thermo Scientific, West Palm Beach, USA).

### One-step competitive indirect ELISA

**Fig. 1:**
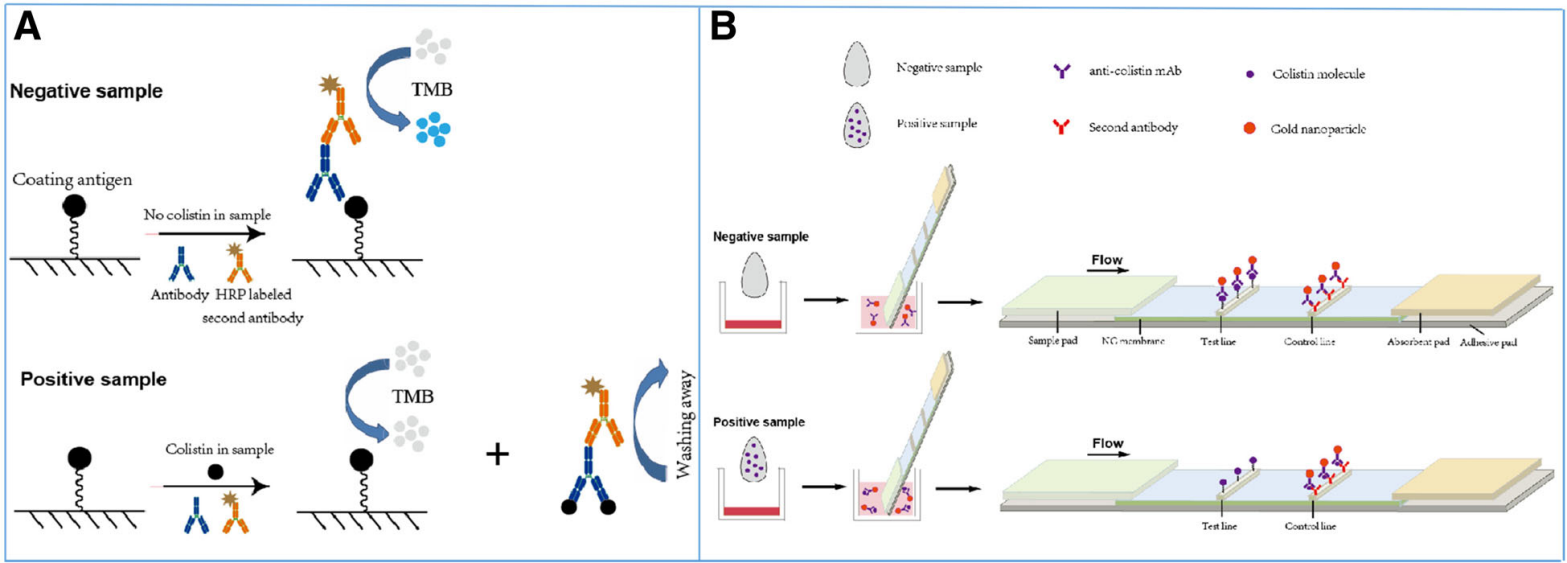
Schemes of one-step competitive indirect ELISA (**a**) and lateral flow immunochromatographic assay (**b**) for colistin

The assay procedures of one-step ci-ELISA (Fig. 1a) were as follows: Firstly, 100 μL of colistin-OVA conjugate (1.0 μg/mL) was coated on each polystyrene plate well. After incubation at 37 °C for 2 h, the solution in wells was discarded and the plates were then washed three times with 0.01 mol/L PBS containing 0.05% Tween 20 (PBST). Afterwards, 50 μL of analyte was added followed by the addition of a mixture (1:1) of anti-colistin mAb solution (1:2000 in 0.01 mol/L PBS) and HRP labeled goat anti-mouse IgG (1:3000 in 0.01 mol/L PBS). After incubation at 37 °C for 45 min, the plate wells were repeatedly washed with PBST for three times. Then 100 μL of the TMB/H_2_O_2_ substrate/chromogen solution (pH 5.5) was added into each well and was incubated at 37 °C for 10 min. Subsequently, the enzymatic reaction was terminated by adding 50 μL/well of 2 N H_2_SO_4_. Finally, the optical density (OD) was determined at 450 nm by a Synergy™ H4 Hybrid Microplate Reader (BioTek Instruments Inc., USA). A calibration curve was established by plotting relative absorbance (B/B_0_) against the logarithm of colistin concentration and it was then fitted to a four-parameter logistic equation. The IC_50_ value was obtained from the equation and it was used for the evaluation of ELISA sensitivity. The cross-reactivity of several common antimicrobials drugs such as bacitracin, aminoglycosides, β-lactams, tetracyclines, sulfonamides, and quinolones to the colistin ELISA were measured and the cross-reactivity values were calculated according to the following equation: percent cross-reactivity = (IC_50_ of colistin / IC_50_ of analytes × 100%).

### LFIA development

#### Preparation of gold nanoparticle (GNP)

Different sizes of GNPs were prepared according to the following procedures [30]. One hundred milliliter of chlorauric acid solution (0.01%, *w*/*v*) was prepared and then heated to boiling. Subsequently, 2.5, 2.0, 1.6 or 1 mL of 1% (*w*/*v*) sodium citrate solution were added to react with chlorauric acid for 15 min under boiling and stirring conditions. Then the solution was cooled down and deionized water was supplemented to the original volume. Finally, the synthesized GNPs were scanned by ultraviolet (UV) spectrophotometer, and the particle sizes of GNPs were measured by transmission electron microscopy (TEM, JEOL Inc., MA, USA).

#### Preparation of detection reagent

The detection reagent was prepared by conjugating anti-colistin mAb onto the surface of GNPs. Under mild stirring condition, the pH value of gold nanoparticle (10 mL) was adjusted to 8.0 with 0.1 mol/L K_2_CO_3_ solution, then 30 μg of the purified anti-colistin mAb was drop-wise added. Following incubation at room temperature for 20 min, 1 mL of 1% BSA solution was added and the mixed solution was then incubated for 15 min. Subsequently, the prepared Ab-GNP conjugate was centrifuged at 8000 r/min for 15 min, the supernatant was discarded and the precipitate was re-suspended in 10 mL of 0.01 mol/L PBS containing 1% sucrose, 1% BSA and 0.5% Triton X-100 (pH 7.4). Finally, 50 μL of the Ab-GNP conjugate solution was added into each microplate well and was then freeze-dried for use.

#### Immobilization of capture reagent and control reagent

For this LFIA, nitrocellulose membrane with width of 4 cm was used. The goat anti-mouse IgG (0.4 mg/mL) and colistin-OVA (0.25 mg/mL) were dispensed onto the nitrocellulose membrane as the control line and test line, respectively, with dispensing volume of 1 μL/cm line. Then the dispensed nitrocellulose membrane was dried at 37 °C for 6 h and stored under dry conditions until use.

#### Strip assembly

The LFIA strip includes four parts: nitrocellulose membrane, sample pad, absorbent pad, and adhesive backing card (Fig. 1b). The strip assembly was described as below: Briefly, the nitrocellulose membrane lined with capture antigens and control reagent was pasted on the middle of adhesive backing card, then the sample pad and the absorbent pad were pasted on the two ends of adhesive backing card and overlapped the nitrocellulose membrane by 2 mm. Lastly, the assembled plate was cut to strips with width of 4 mm.

#### Test procedure

Briefly, 200 μL of sample solution was added into the micro-plate well containing detection reagent. After dissolving the detection reagent and incubating for 3 min, the sample pad end of test strip was immersed into the well, and then the solution would flow toward the absorbent pad. After 10 min, the test result can be visually judged, and the line intensities can be measured by a strip reader (NB Gene Ltd., Beijing, China).

### Sample preparation and analysis

The sample preparation procedures are described as below which referred to the procedures from literature report [15, 24]. For animal feed sample, 1.0 g of the sample was weighed and added with 100 mL of 2% trichloroacetic acid aqueous solution (TCA) containing 1% Triton X-100. Then the mixed solution was continually stirred for 30 min and was subsequently filtered to obtain the supernatant. The pH value of the extract was adjusted to neutral and the solution was ready for ELISA or LFIA analysis. For milk sample, 1.0 g of the sample was mixed with 4 mL of 2% TCA aqueous solution containing 1% Triton X-100 and was then vortexed for 5 min. Subsequently, the mixed solution was filtered and the solution pH value was adjusted to neutral. Finally, the neutralized solution was applied to ELISA or LFIA analysis. For pork meat sample, 1.0 g of the sample was weighed and mixed with 20 mL of 4% TCA aqueous solution containing 1% Triton X-100). The mixed solution was ultrasonic-assisted extracted for 20 min and was then filtered to obtain the supernatant. After the solution was neutralized with sodium hydroxide, it was then subjected to ELISA or LFIA analysis. For spiked experiment, colistin standard solution was added into blank samples and the spiked concentrations were 0.5–4.0 mg/kg for swine feed and chicken feed samples, 25–100 μg/kg for milk samples and 75–300 μg/kg for meat samples, respectively. Four different blank samples for each type of sample matrix were used in the spiked experiment. The spiked samples and the blank samples were then pretreated and analyzed by the developed ELSA and LFIA. Three repeats were performed for each sample. For real sample analysis, 20 samples were simultaneously measured by the developed immunoassays and LC-MS/MS method [24] and their analytical results were compared.

## Results and discussion

### Antibody preparation and characterization

**Fig. 2:**
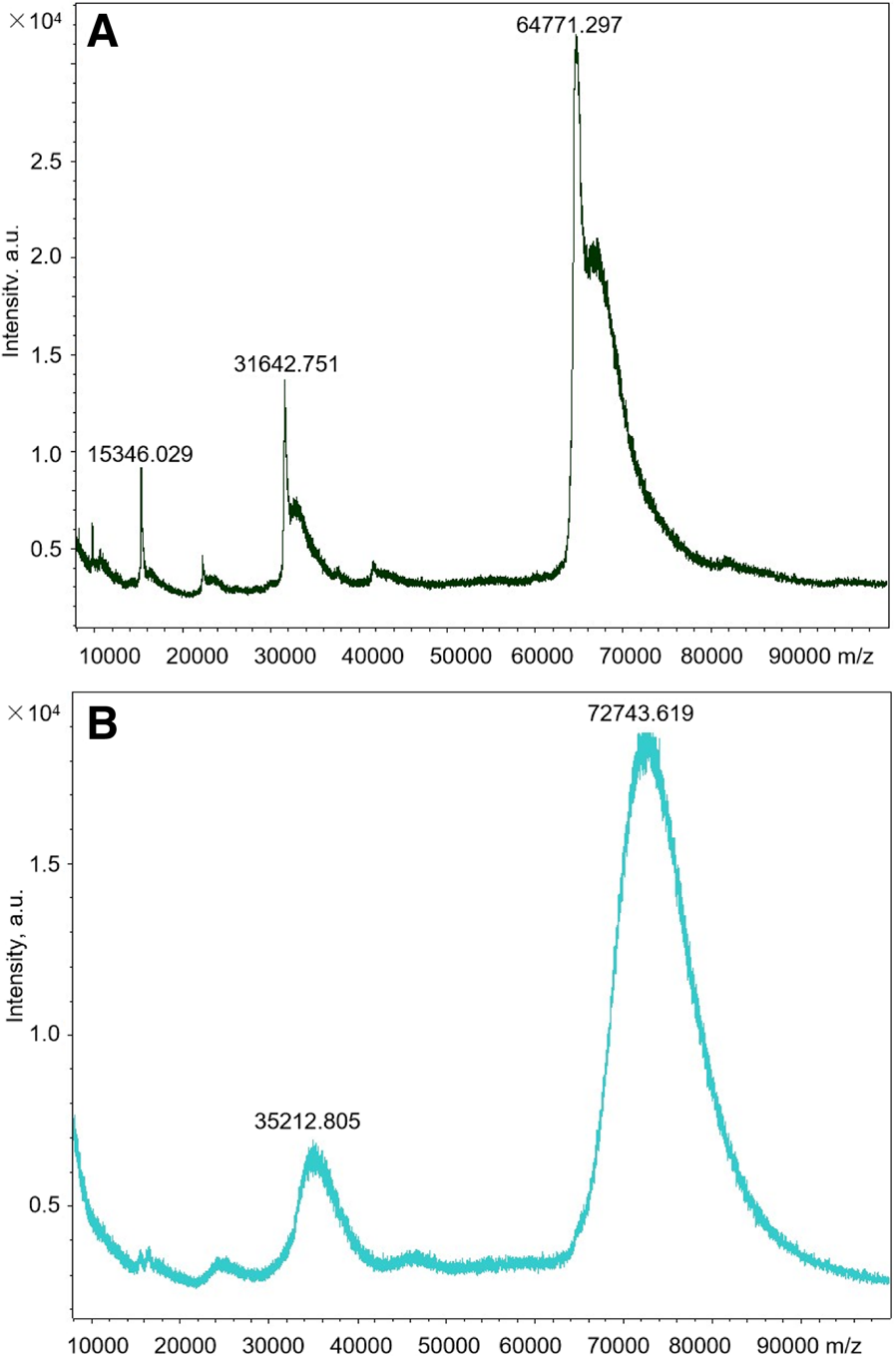
MALDI-TOF analysis of BSA (**a**) and colistin-BSA conjugate (**b**)

Colistin is a small molecule which cannot elicit animal body to produce a specific antibody, thus it was covalently linked with BSA by EDC coupling method to form an immunogen for antibody preparation. By MALDI-TOF analysis, the conjugation ratio of colistin to BSA is about 6.9:1 (Fig. 2), which was considered to be suitable for antigen immunization and antibody production [31]. The antibody titer and affinity of sera from mice was then monitored since the third immunization. The result indicated that the titers and IC_50_ values of all sera remained roughly the same between the third and the fourth immunization, therefore, after the fifth immunization, the final bleeding was performed. For investigating the effect of immunization dosage on antibody affinity, two dosage groups (15 μg and 60 μg per immunization) were performed in this study. The low dosage group led to IC_50_ values in the range of 68 to 133 ng/mL, while the high dosage group resulted in IC_50_ values of 76 to 149 ng/mL (Additional file 1: Table S1), indicating that the two immunization dosages did not produce significant difference on the affinities of sera antibodies. The mouse from low-dosage group produced the serum with the lowest IC_50_ value of 68 ng/mL, and it was then sacrificed for hybridoma preparation. After cell fusion and cloning, four hybridomas excreting anti-colistin antibodies were obtained. The hybridoma named 3D5-E7 was chosen for antibody production because it can excrete mAb with the lowest IC_50_ value of 10.1 ng/mL. This means that as compared to the mouse antiserum, the affinity of this mAb increased by about 7 times, thus the mAb from the hybridoma 3D5-E7 was further evaluated for antibody specificity and was used for subsequent immunoassay development. The isotype of this mAb was determined to be IgG 2a. Several common antimicrobial drugs such as bacitracin, aminoglycosides, β-lactams, tetracyclines, sulfonamides, quinolones, and chloramphenicol were tested for antibody specificity. The result showed that this mAb can specifically recognized colistin and it had negligible cross-reactivity with other antimicrobial drugs as mentioned above (Table 1).

**Table 1:** Cross-reactivity of anti-colistin mAb with other common antibacterial drugs

Analyte	IC_50_, ng/mL	Cross-reactivity, %
Colistin	10.1	100
Bacitracin	> 2000	<0.1
Neomycin	> 2000	<0.1
Penicillin G	> 2000	<0.1
Tetracycline	> 2000	<0.1
Sulfadimidine	> 2000	<0.1
Enrofloxacin	> 2000	<0.1
Chloramphenicol	> 2000	<0.1

### ELISA optimization

**Fig. 3:**
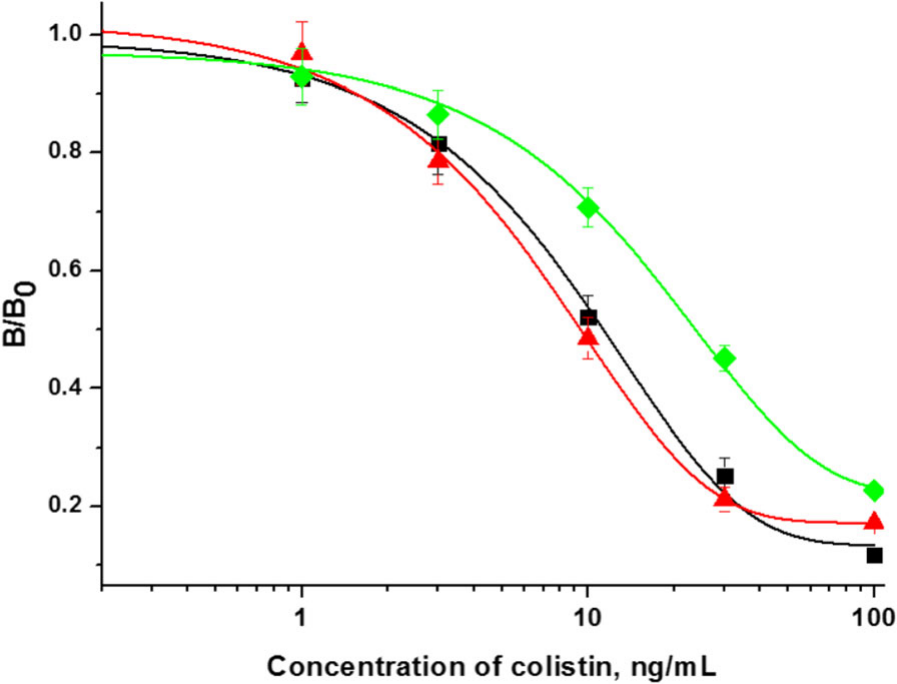
Calibration curves of three types of ELISA for colistin (*n* = 4): (■) One-step ci-ELISA; (▲) Two-step ci-ELISA; (◆) cd-ELISA

Conventional ELISA generally employs competitive direct ELISA (cd-ELISA) or ci-ELISA format (Additional file 1: Figure S1). The cd-ELISA only needs one-step immunological reaction, but it requires the preparation of hapten-enzyme conjugate. The ci-ELISA format employs commercially available second antibody-enzyme conjugate, but it normally requires two-step immunological reaction. In this study, we introduced a one-step ci-ELISA and compared this assay format with cd-ELISA and two-step ci-ELISA regarding assay sensitivity and assay time (Additional file 1: Table S2). After the optimization of pH value, ionic strength, coating condition, and incubation time for each step, the assay performance of the three assay formats was shown in Fig. 3. The result indicated that the assay sensitivities of one-step ci-ELISA (IC_50_ = 9.7 ng/mL) and two-step ci-ELISA (IC_50_ = 9.1 ng/mL) were comparable, and both of them are superior to cd-ELISA (IC_50_ = 23.7 ng/mL). As the assay time of one-step ci-ELISA (~ 60 min) was much less than two-step ci-ELISA (~ 110 min), the newly developed one-step ci-ELISA was selected to perform in the subsequent experiment.

### Optimization of LFIA

#### The effect of gold nanoparticle

To study the effect of GNP size on LFIA sensitivity, four sizes of GNPs were synthesized [30]. The UV-vis spectra of the four GNPs solutions synthesized by reducing chlorauric acid with 1.0, 1.6, 2.0 and 2.5 mL of citrate solution showed maximum absorption peaks at 530, 526, 522 and 519 nm, respectively (Additional file 1: Figure S2). Transmission electron microscope (TEM) images of the four GNPs are shown in Additional file 1: Figure S3 and their sizes were measured to be 36, 25, 16 and 12 nm, respectively. Literature reports indicated that larger size of GNPs can produce higher assay sensitivity than smaller GNPs [32, 33]. In our study, 36 nm GNPs also result in slightly higher sensitivity than other sizes of GNPs. However, it was found that the Ab-GNPs conjugate with 36 nm GNPs was not stable and tended to aggregation during sample preparation process, which may be attributed to the high salt concentration in sample extracts. In contrast, the Ab-GNP conjugate prepared with smaller GNPs was much more stable. Therefore, we finally chose 25 nm of GNPs for the LFIA development.

### Optimizing pH value and antibody amount for Ab-GNP preparation

Antibodies can physically conjugate on the surface of GNPs by electrostatic interaction. The pH value and antibody amount for Ab-GNP preparation would influence the antibody orientation and density on GNP [33], and thus both parameters may be related to the affinity of Ab-GNP conjugate and affect the LFIA sensitivity. Hence a checkerboard test was performed to select the appropriate pH value and the antibody amount for the preparation of Ab-GNP. The result indicated that the increase of antibody amount for Ab-GNP preparation produces stronger test line intensity, while the strength of test line intensity becomes weaker with the increase of pH value. At similar line intensity, the optimal combination of pH value and antibody amount that led to the most significant difference between negative and positive samples were determined to be pH 8.0 and 3.0 μg of anti-colistin mAb, respectively.

### Reduction of non-specific adsorption

**Fig. 4:**
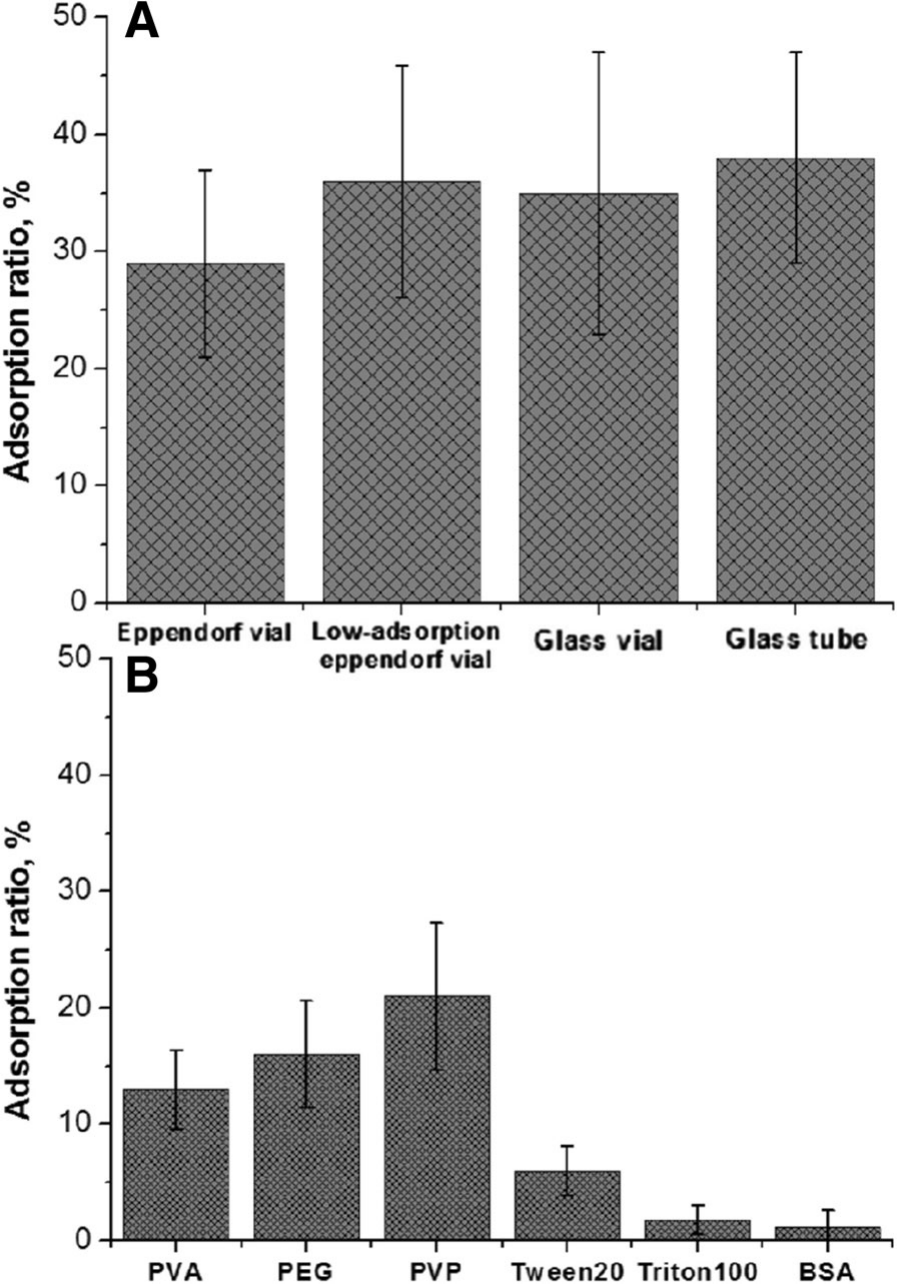
Adsorption ratio of colistin in different vials (**a**) and after blocking with different reagents (**b**)

The non-specific adsorption of colistin on vials is one major problem for colistin analysis, which could result in the low recovery of colistin from samples. In order to reduce the effect of non-specific adsorption, most instrumental analysis employed polymyxin B as internal standard [23, 27]. Because polymyxin B antibiotic is an analogue of colistin which can also be recognized by anti-colistin mAb, it cannot be used to verify the assay accuracy of colistin immunoassays. To address the non-specific adsorption problem, we firstly tested the adsorption ratios of colistin on different vials with different materials including Eppendorf vial, low-adsorption Eppendorf vial, and glass vial. Unfortunately, colistin can absorb on all the tested vials (Fig. 4a). Then we tried several polymers including polyvinyl pyrrolidone (PVP), polyethylene glycol (PEG), and polyvinyl alcohol (PVA) to block the non-specific adsorption sites on vials. Although the non-specific adsorption of colistin can be partly reduced by these polymers, the non-specific problem was still significant. (Fig. 4b). Literature has reported the use of BSA to reduce colistin adsorption in urine sample [23], and we also confirmed that the addition of BSA in standard colistin solution could effectively reduce the non-specific adsorption (Fig. 4b). However, our further experiment found that colistin can also produce non-specific adsorption in acid sample extraction solution (2% TCA). At acid condition, BSA would be denatured and form precipitate after centrifugation during sample pretreatment process, and hence it cannot be used in acid extraction solution. As an alternative, we then tested the surfactants including Tween 20 and Triton X-100 for blocking the non-specific adsorption (Fig. 4b). As a result, Triton X-100 presented a good effect on reducing non-specific adsorption, and the concentration of 1% presented the best effect, thus we chose 1% Triton X-100 to be used as a blocking reagent which was added into the sample extraction solution and standard colistin solution to eliminate the non-specific adsorption.

### Method validation

#### One-step ci-ELISA

The limits of detection (LOD) are defined as 3.3 Ϭ/S, where Ϭ is the standard deviation of background signal from blank samples, and S is the slope of calibration curve [34]. By calculation, the LOD of the one-step ELISA were measured to be 101.4 μg/kg for swine feed sample, 103.2 μg/kg for chicken feed sample, 5.4 μg/kg for milk sample and 24.6 μg/kg for meat samples (*n* = 20), respectively. At the spiked colistin concentrations of 1.0–4.0 mg/kg in swine feed and chicken feed, 25–100 μg/kg in milk sample, and 75–300 μg/kg in meat sample, the corresponding recoveries ranged from 81.63–108.00%, 87.44–110.13%, and 77.83–97.52%, respectively, with coefficients of variability (CVs) less than 12.16% (Table 2), indicating that the accuracy and precision of this ELISA meet the requirement of quantitative analysis. Compared to the previously reported ELISA methods (Additional file 1: Table S3), the assay sensitivity of this ELISA is much higher than that of Kitagawa et al. [25] and its assay accuracy is better than that of Suhren and Knappstein [15].

**Table 2:** Spiked recoveries and coefficient of variations (CVs) of one-step ci-ELISA and LFIA for colistin in animal feed and food (*n* = 4)

Method	Sample type	Spiked concentration	Measured concentration	Recovery, %	CV, %
One-step ELISA	Swine feed, mg/kg	0	< LOD	–	–
		1.0	0.93 ± 0.11	93.20	11.40
		2.0	1.86 ± 0.18	93.03	9.53
		4.0	3.27 ± 0.08	81.63	2.48
	Chicken feed, mg/kg	0	< LOD	–	–
		1.0	1.08 ± 0.09	108.00	8.33
		2.0	1.93 ± 0.14	96.57	7.30
		4.0	3.89 ± 0.18	97.34	4.56
	Milk, μg/kg	0	< LOD	–	–
		25	27.53 ± 2.97	110.13	10.78
		50	49.87 ± 3.41	99.74	6.83
		100	87.44 ± 7.79	87.44	8.91
	Meat, μg/kg	0	< LOD	–	–
		75	73.14 ± 8.89	97.52	12.16
		150	116.75 ± 13.13	77.83	11.25
		300	253.17 ± 24.79	84.39	9.79
LFIA	Swine feed, mg/kg	0	< LOD	–	–
		0.5	0.44 ± 0.07	87.86	16.75
		1.0	1.14 ± 0.16	114.45	13.94
		2.0	1.74 ± 0.15	87.23	8.57
	Chicken feed, mg/kg	0	< LOD	–	–
		0.5	0.56 ± 0.05	112.84	9.04
		1.0	0.95 ± 0.13	95.43	13.57
		2.0	1.92 ± 0.12	95.85	6.03
	Milk, μg/kg	0	< LOD	–	–
		25	28.35 ± 3.79	113.38	13.37
		50	40.59 ± 5.26	81.17	12.96
		100	89.93 ± 8.96	89.93	9.96
	Meat, μg/kg	0	< LOD	–	–
		75	74.45 ± 13.37	99.27	17.96
		150	150.72 ± 20.36	100.48	13.51
		300	234.66 ± 34.52	78.22	14.71

#### LFIA

**Fig. 5:**
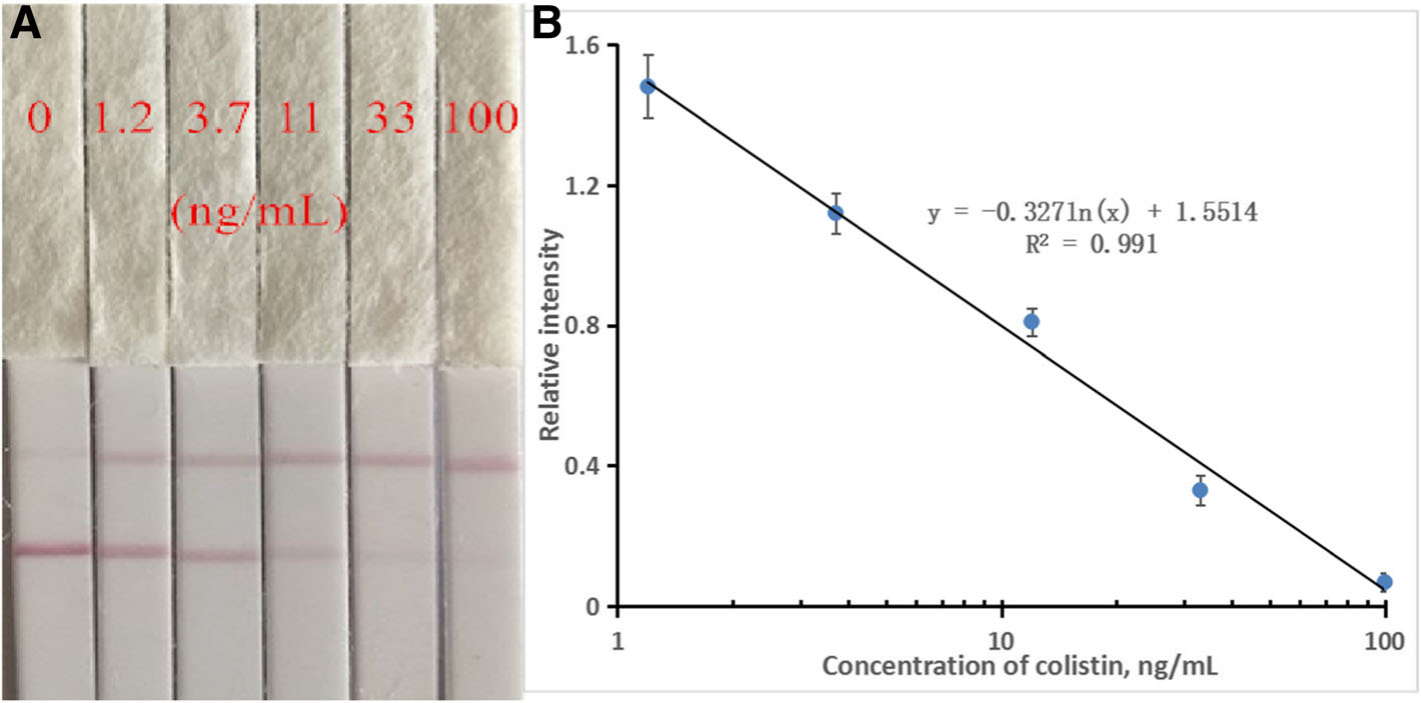
Typical photo image (**a**) and calibration curve (**b**) of LFIA for different concentrations of colistin (*n* = 4)

The typical photo images of LFIA strip test are illustrated in Fig. 5. For semi-quantitative analysis, the standard curve was established using the relative optical density of the spiked and blank sample (B/B_0_) as the Y axis and the logarithmic concentration of colistin as the X axis (Fig. 5). The limit of detection (LOD) was set as the concentration of the analyte that leads to a 20% reduction in test line intensity as compared to blank sample. The LODs of colistin in phosphate buffer was calculated as 0.87 ng/mL. By multiplying dilution factors, the LODs of colistin in swine feed, chicken feed, milk and meat were determined to be 110.4, 118.7, 5.9 and 23.9 μg/kg, respectively, which were close to that of the developed ELISA method and were far below than the generally addition content of colistin (mg/kg level) in animal feed [24] and the MRLs (50 or 150 μg/kg) in animal-origin food [12]. To evaluate the capability of these LODs to distinguish negative and positive sample, various blank samples (four different samples for each matrix and three repeats for each sample) were subjected to the LFIA. It was indicated that the calculated detection values were all below the established LODs, demonstrating that these LODs would not result in false positive results (Table 2). Furthermore, the spiked recovery experiment was used to evaluate the LFIA accuracy and precision. As shown in Table 2, at the spiked concentration, the corresponding recoveries of colistin from animal feed, milk and meat samples ranged from 87.23–114.45%, 81.17–113.38% and 78.22–100.48% respectively with CVs less than 18.26%, demonstrating that the developed LFIA can be used as semi-quantitative analysis.

#### Analysis of real samples

**Fig. 6:**
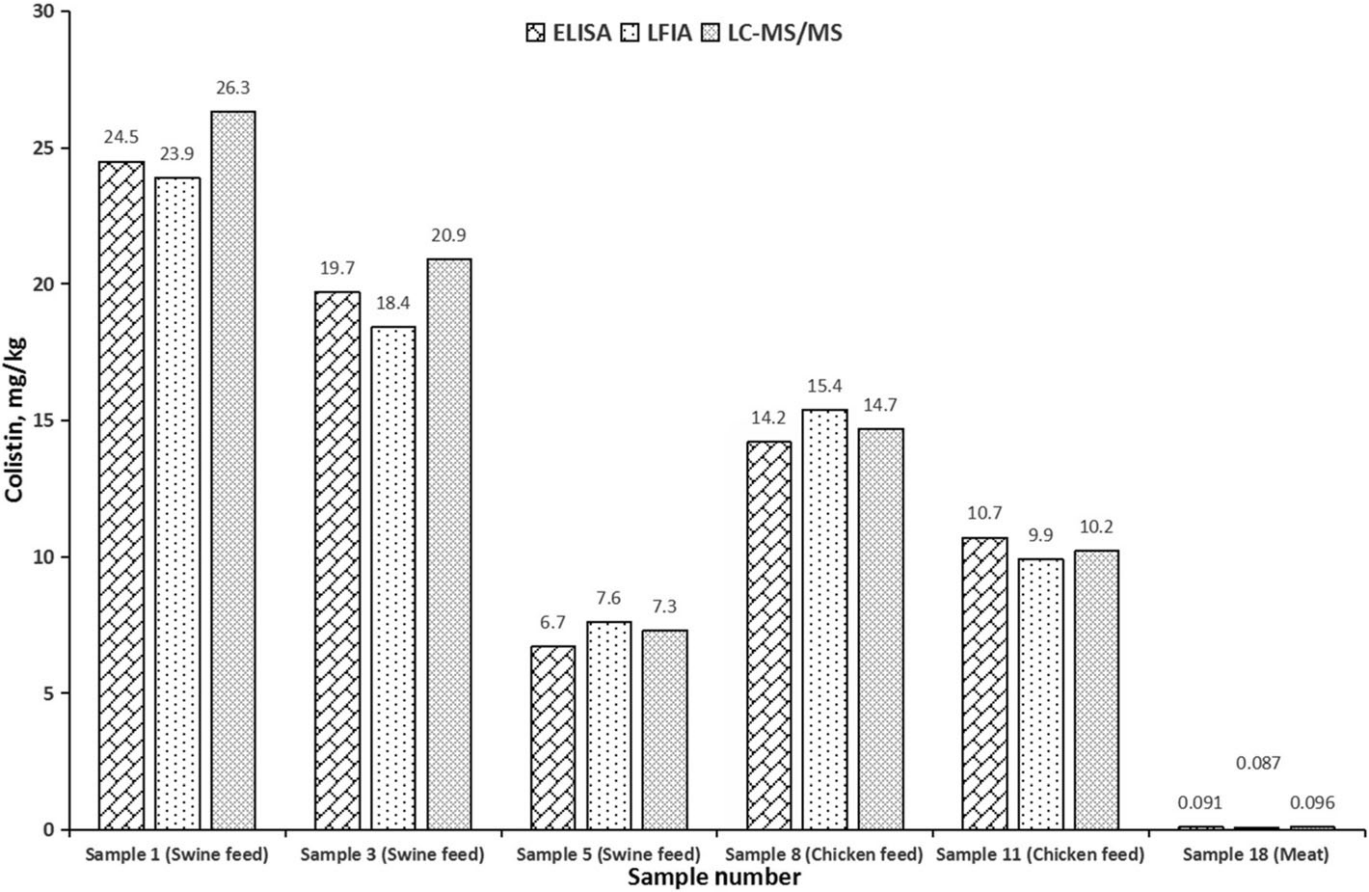
Detection results of colistin in actual positive samples by ELISA, LFIA and HPLC method

To further validate the developed assays, twenty actual animal feed and food samples (six swine feed samples, six chicken feed samples, four milk samples and four meat samples) were collected from different farms and local markets and were analyzed by the developed ELISA, LFIA and LC-MS/MS methods. It should be noted that the feed samples were obtained from local market before the prohibited use of colistin in feed. As shown in Additional file 1: Table S4, fourteen samples including three swine feed samples, four chicken feed samples, four milk samples and three meat samples were determined as negative (Additional file 1: Table S4) by both the developed immunoassays and LC-MS/MS method. This result indicated the developed immunoassays did not produce false negative result. Six samples including three swine feed samples, two chicken feed samples and one meat sample were measured to contain colistin, and the detection values obtained by the developed ELISA and LFIA were consistent with that of LC-MS/MS method (Fig. 6). These results demonstrated that the developed ELISA and LFIA could be used for the accurate determination of colistin in actual samples.

## Conclusion

In the study, we developed a rapid one-step ci-ELISA and a LFIA for colistin in animal feed and food. The LODs of both methods are far below the generally addition content of colistin (mg/kg level) in animal feed and also meet the MRLs (50 or 150 μg/kg) in animal-origin food set by authorities. Also, the recoveries of colistin from animal feed and food were within acceptable range with good assay precision. Furthermore, the analysis time of the one-step ci-ELISA was less than 60 min, and the analysis time of LFIA was less than 15 min. Thus, these two immunoassays can be selectively used for rapidly monitoring the illegal use of colistin in animal feed and the colistin residue in animal-origin food.

## Additional file

Additional file 1: **Figure S1.** Schemes of one-step indirect ELISA, two-step indirect ELISA and one-step direct ELISA for colistin. **Figure S2.** Ultraviolet-visible spectrum of four GNPs solutions synthesized by reducing chlorauric acid with 1.0, 1.6, 2.0 and 2.5 mL of citrate solution. **Figure S3.** TEM images of four GNPs solutions synthesized by reducing chlorauric acid with 1.0 mL (A), 1.6 mL (B), 2.0 mL (C) and 2.5 mL (D) of citrate solution. **Table S1.** The IC_50_ values of all sera from mice immunized with two different dosages. **Table S2.** Comparison of one step ci-ELISA, two step ci-ELISA and one step cd-ELISA regarding IC_50_ values and assay time (*n* = 4). **Table S3.** Comparison of the developed ELISA method with that literature reported enzyme immunoassay. **Table S4.** Detection results of colistin in actual animal feed by ELISA, LFIA and HPLC method. (DOCX 723 kb)

## Data Availability

All data generated or analyzed during this study are included in this published article.

## Abbreviations

BSA: Bovine serum albumin
ci-ELISA: Competitive indirect ELISA
CVs: Coefficients of variability
EDC: 1-Ethyl-3-(3-dimethylaminopropyl) carbodiimide
ELISA: Enzyme-linked immunosorbent assay
FBS: Fetal bovine serum
FMOC-Cl: 9-Fluorenylmethyl chloroformate
GNP: Gold nanoparticle
HAT: Hypoxanthine-aminopterin-thymidine
HPLC: High performance liquid chromatography
HRP: Horseradish peroxidase
HT: Hypoxanthine-thymidine
IC_50_: Half maximal inhibitory concentration
IgG: Immunoglobulin G
LC-MS/MS: Liquid chromatography coupled with tandem mass spectrometry
LFIA: Lateral flow immunochromatographic assay
LOD: The limits of detection
mAb: Monoclonal antibody
MRLs: Maximum residue limits
OD: The optical density
OVA: Ovalbumin
PBS: Phosphate buffer saline
PEG 1500: Polyethylene glycol 1500
PEG: Polyethylene glycol
PVA: Polyvinyl alcohol
PVP: Polyvinyl pyrrolidone
TCA: Trichloroacetic acid aqueous solution
TEM: Transmission electron microscope
UV: Ultraviolet
