# Supplementary material for: Rapid one-step enzyme immunoassay and lateral flow immunochromatographic assay for colistin in animal feed and food
Source: J Anim Sci Biotechnol. 2019 Oct 17;10:82. doi: 10.1186/s40104-019-0389-7 (PMC6796504; doi:10.1186/s40104-019-0389-7)
Supplement: Supplementary file 1 — Figure S1. Schemes of one-step indirect ELISA, two-step indirect ELISA and one-step direct ELISA for colistin. Figure S2. Ultraviolet-visible spectrum of four GNPs solutions synthesized by reducing chlorauric acid with 1.0, 1.6, 2.0 and 2.5 mL of citrate solution. Figure S3. TEM images of four GNPs solutions synthesized by reducing chlorauric acid with 1.0 mL (A), 1.6 mL (B), 2.0 mL (C) and 2.5 mL (D) of citrate solution. Table S1. The IC50 values of all sera from mice immunized with two different dosages. Table S2. Comparison of one step ci-ELISA, two step ci-ELISA and one step cd-ELISA regarding IC50 values and assay time (n = 4). Table S3. Comparison of the developed ELISA method with that literature reported enzyme immunoassay. Table S4. Detection results of colistin in actual animal feed by ELISA, LFIA and HPLC method. (DOCX 723 kb) [file 40104_2019_389_MOESM1_ESM.docx]

Supplementary material

**Rapid One-step Enzyme Immunoassay and Lateral Flow Immunochromatographic Assay for Colistin in Animal Feed and Food**

Jiayi Wang ^1^, Jinyu Zhou ^1^, Yiqiang Chen ^1, *^, Xinpei Zhang ^1^, Yongpeng Jin ^1^, Xiaoqian Cui ^1^, Dongting He ^1^, Wenqing Lai ^1^, Lidong He ^2^

^1^ *Beijing Advanced Innovation Center for Food Nutrition and Human Health, and State Key Laboratory of Animal Nutrition, College of Animal Science and Technology, China Agricultural University, Beijing, China*

^2^ *Department of Chemistry and Biochemistry, Florida State University, Tallahassee, FL, USA*

**^*^ Corresponding author:**

Yiqiang Chen, Ph. D, Associate Professor

Tel: (86-10)62733764; Fax: (86-10)62733588; E-mail: [yqchen@cau.edu.cn](mailto:yqchen@cau.edu.cn)


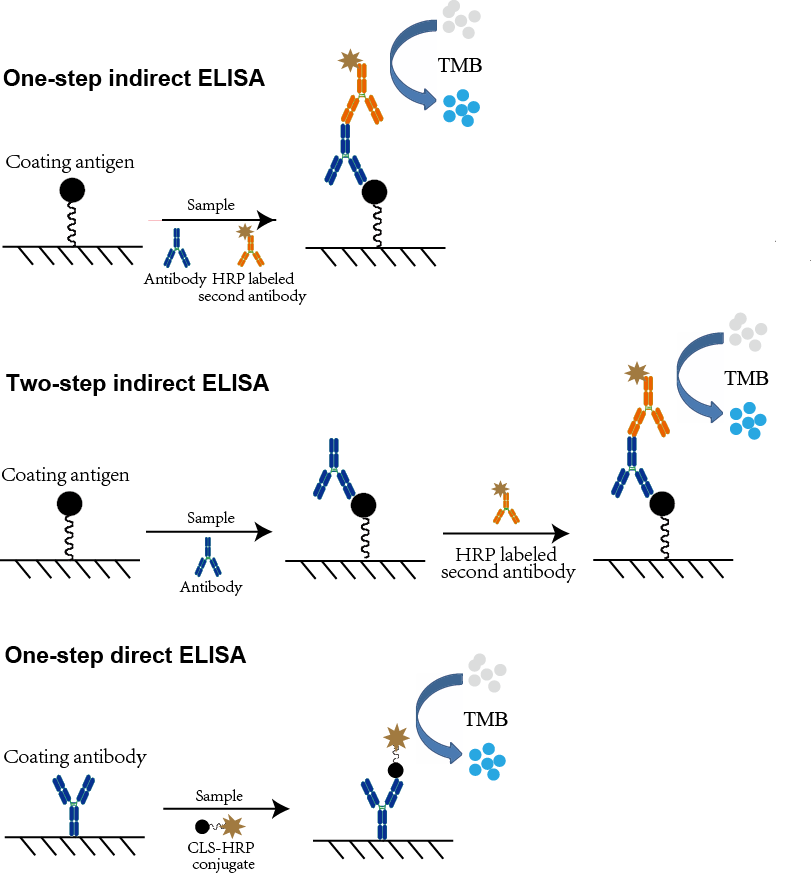


**Fig. S1** Schemes of one-step indirect ELISA, two-step indirect ELISA and one-step direct ELISA for colistin.


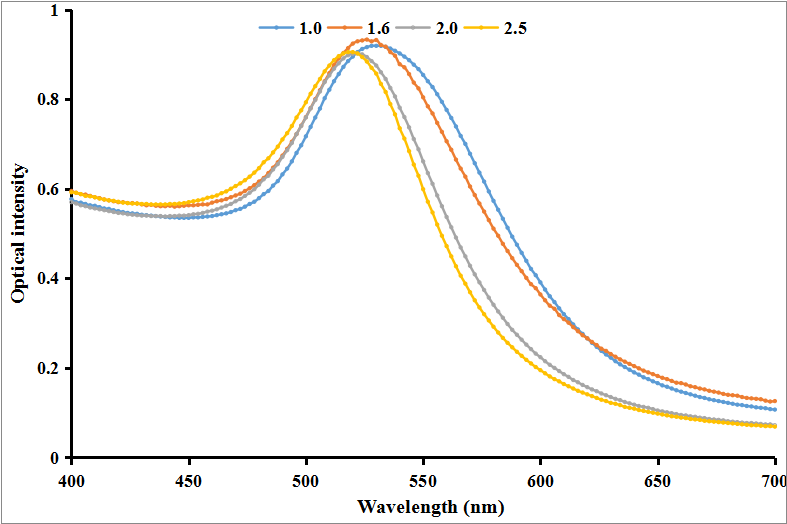


**Fig. S2** Ultraviolet-visible spectrum of four GNPs solutions synthesized by reducing chlorauric acid with 1.0, 1.6, 2.0 and 2.5 mL of citrate solution.


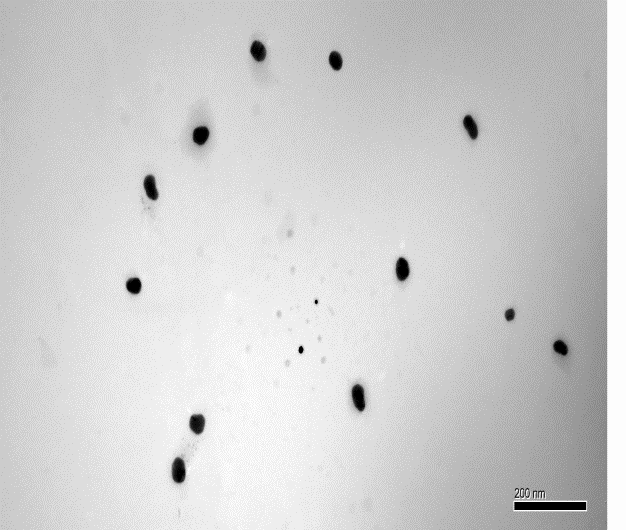

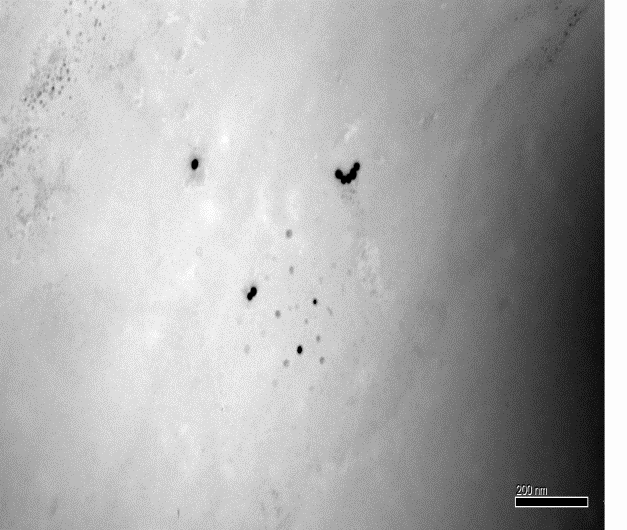

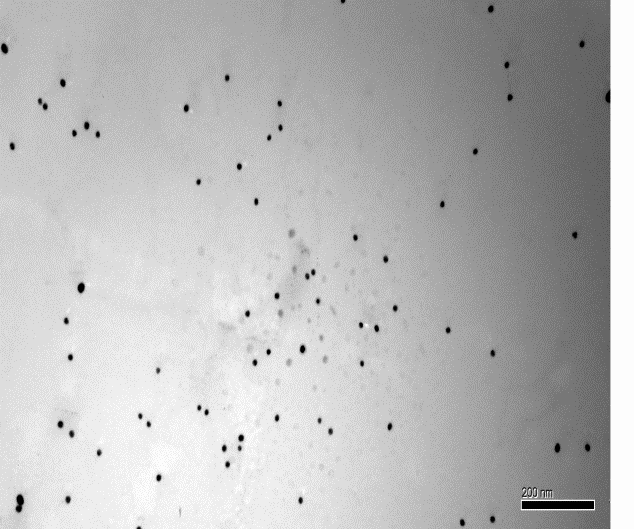

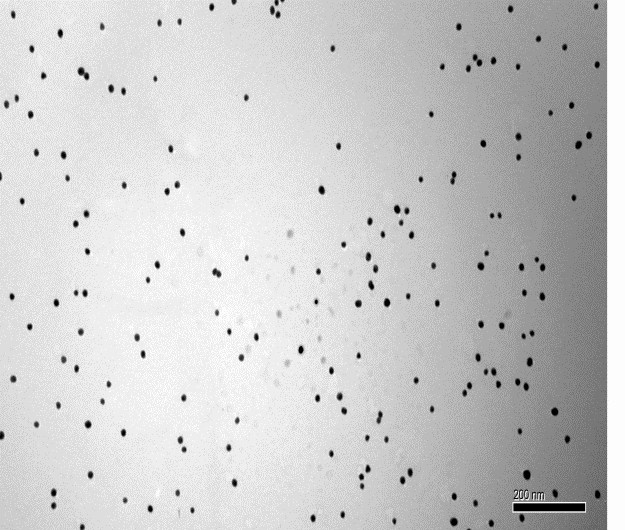


1. **A**
2. **B**
3. **C**
4. **D**

**Fig. S3** TEM images of four GNPs solutions synthesized by reducing chlorauric acid with 1.0 mL (A), 1.6 mL (B), 2.0 mL (C) and 2.5 mL (D) of citrate solution.

**Table S1** The IC_50_ values of all sera from mice immunized with two different dosages.

| **Group 1** | **1** | **2** | **3** | **4** | **5** | **6** | **7** | **8** | **9** | **10** |
| --- | --- | --- | --- | --- | --- | --- | --- | --- | --- | --- |
| IC_50_ values (ng/mL) | 82 | 111 | 93 | 77 | 68 | 85 | 116 | 133 | 121 | 89 |
| **Group 2** | **1** | **2** | **3** | **4** | **5** | **6** | **7** | **8** | **9** | **10** |
| IC_50_ values (ng/mL) | 124 | 149 | 88 | 95 | 101 | 79 | 76 | 112 | 86 | 92 |

Note: Group 1: Low dosage group, 15 μg per immunization; Group 2: High dosage group, 60 μg per immunization.

**Table S2** Comparison of one step ci-ELISA, two step ci-ELISA and one step cd-ELISA regarding IC_50_ values and assay time (n=4).

|  | One step ci-ELISA | two step ci-ELISA | one step cd-ELISA |
| --- | --- | --- | --- |
| IC_50_ value (ng/mL) | 9.7±0.2 | 9.1±0.1 | 23.7±0.4 |
| Assay time | ~60 min | ~110 min | ~60 min |

**Table S3** Comparison of the developed ELISA method with that literature reported enzyme immunoassay.

| Limit of detection | Assay accuracy | Assay precision | Literature |
| --- | --- | --- | --- |
| 30 ng/mL in buffer | --- | --- | Kitagawa et al., 1985 |
| 10.9 μg/kg in milk | 116-146% | 10.8-56.2% | Suhren and Knappstein, 2005 |
| 5.9 μg/kg in milk | 87.44-110.13%, | 8.91-10.78% | This study |

**Table S4** Detection results of colistin in actual animal feed by ELISA, LFIA and HPLC method.

| Sample No. | 1 | 2 | 3 | 4 | 5 | 6 | 7 | 8 | 9 | 10 |
| --- | --- | --- | --- | --- | --- | --- | --- | --- | --- | --- |
| Sample type | Swine feed | Swine feed | Swine feed | Swine feed | Swine feed | Swine feed | Chicken feed | Chicken feed | Chicken feed | Chicken feed |
| ELISA, mg/kg | 24.5 | － | 19.7 | － | 6.7 | － | － | 14.2 | － | － |
| LFIA, mg/kg | 23.9 | － | 18.4 | － | 7.6 | － | － | 15.4` | － | － |
| LC-MS, mg/kg | 26.3 | － | 20.9 | － | 7.3 | － | － | 14.7 | － | － |
| Sample No. | 11 | 12 | 13 | 14 | 15 | 16 | 17 | 18 | 19 | 20 |
| Sample type | Chicken feed | Chicken feed | Milk | Milk | Milk | Milk | Meat | Meat | Meat | Meat |
| ELISA, mg/kg | 10.7 | － | － | － | － | － | － | 0.091 | － | － |
| LFIA, mg/kg | 9.9 | － | － | － | － | － | － | 0.087 | － | － |
| LC-MS, mg/kg | 10.2 | － | － | － | － | － | － | 0.096 | － | － |
